# Biomechanical index for predicting the risk of acute coronary syndrome

**DOI:** 10.3389/fcvm.2026.1766059

**Published:** 2026-03-24

**Authors:** Michael J. Johnson, Michael R. A. Abdelmalik, Gilwoo Choi, Shaolie S. Hossain, Bon-Kwon Koo, Sabrina R. Lynch, Kersten Petersen, Gregory J. Rodin, Michiel Schaap, Charles A. Taylor, Christopher K. Zarins, Thomas J. R. Hughes

**Affiliations:** 1 Oden Institute, University of Texas, Austin, TX, United States; 2 Department of Mechanical Engineering, Eindhoven University of Technology, Eindhoven, Netherlands; 3 HeartFlow, Inc., Mountain View, CA, United States; 4 Molecular Cardiology Research Laboratory, The Texas Heart Institute, Houston, TX, United States; 5 Department of Internal Medicine and Cardiovascular Center, Seoul National University Hospital, Seoul, Republic of Korea; 6 Department of Internal Medicine, University of Texas, Austin, TX, United States

**Keywords:** biomechanical model, computational medicine, coronary artery disease, coronary computed tomographic angiogram (CCTA), FFR-CT, finite element stress analysis, vulnerable plaque, cap vulnerability index (CVI)

## Abstract

**Background:**

A patient-specific biomechanical model for quantifying the stress state within coronary atherosclerotic lesions for the noninvasive prediction of acute coronary syndrome (ACS) has not been established.

**Methods:**

Sixty-nine patients with clearly documented ACS and available coronary computed tomographic angiography (cCTA) acquired between 1 month and 2 years before the development of ACS were included. In 63 culprit and 146 nonculprit lesions, lumen geometry, the presence of adverse plaque characteristics (APC), and hemodynamic parameters were evaluated using previously described methods. A novel biomechanical metric, denoted as the Cap Vulnerability Index (CVI), was calculated from cCTA data using computational solid and fluid mechanics and compared to anatomic, plaque and hemodynamics variables for the ability to discriminate culprit from nonculprit lesions.

**Results:**

Culprit lesions had a greater diameter stenosis, lesion length, more adverse plaque and hemodynamic characteristics and a greater CVI. Among all parameters evaluated, CVI had the highest AUC (0.75), was the strongest independent predictor for ACS and was comparable to previously described statistical models combining anatomy, plaque and hemodynamic variables.

**Conclusions:**

Noninvasive biomechanical assessment of coronary plaques using computational solid and fluid mechanics may improve identification of culprit lesions for ACS and simplify the interpretation of risk factors for individual lesions.

## 1 Introduction

Acute Coronary Syndrome (ACS) is a critical event that is most commonly caused by plaque rupture (1). The ability to identify coronary plaques that are at high risk of rupture causing subsequent ACS has significant implications for the prognosis and treatment of patients with coronary artery disease.

Previous studies using virtual histology intravascular ultrasound (VH-IVUS) and optical coherence tomography (OCT) have demonstrated clinical value in qualitative and quantitative assessment of the lumen and underlying plaque (2). However, due to relatively low positive predictive values and their invasive nature, these modalities have received limited adoption in the clinical setting (3, 4).

Noninvasive imaging studies using coronary Computed Tomography Angiography (cCTA) have also demonstrated clinical value for qualitative and quantitative lumen and plaque assessment. Motoyama et al. first demonstrated the opportunity to use CCTA-derived “adverse plaque characteristics” to assess risk of myocardial infarction noninvasively (5) clinical utility of hemodynamic assessments using cCTA and computational fluid dynamics (CFD). In the EMERALD (Exploring the MEchanism of plaque Rupture in Acute coronary syndrome using coronary CT Angiography and computationaL fluid Dynamics) study, Lee et al. showed that cCTA enables simultaneous lumen, plaque, and hemodynamic assessments of coronary lesions, thus discriminating between culprit lesions causal of ACS from nonculprit lesions (6, 7). Subsequently, in the DESTINY (PreDiction and Validation of Clinical CoursE of Coronary Artery DiSease With CT-Derived Non-INvasive HemodYnamic Phenotyping and Plaque Characterization, NCT04794868) study Lee et al. again showed the value of combining hemodynamic factors with lesion geometry and plaque characteristics to identify culprit lesions and predict subsequent ACS (8). In the largest and most recent study to examine the role of anatomy, plaque and hemodynamic characteristics in predicting subsequent ACS, the EMERALD II (Exploring the Mechanism of Plaque Rupture in Acute Coronary Syndrome Using Coronary Computed Tomography Angiography and Computational Fluid Dynamics II) trial, Koo et al. described the value of AI-enabled quantitative plaque and hemodynamic characteristics to discriminate between culprit and nonculprit lesions and predict subsequent ACS (9). In all of these studies conducted to date, lesion geometry, plaque characteristics, and hemodynamic forces have been compared against one another to discriminate culprit from nonculprit lesions. In all of these studies, (i) a hemodynamic variable, the change in fractional flow reserve derived from CCTA, or $\Delta {\mathrm{FFR}}_{\mathrm{CT}}$, was the single best parameter to discriminate culprit from nonculprit lesions and (ii) statistical or machine-learning models constructed including anatomy, plaque and hemodynamic factors yielded the best overall performance.

However, machine learning models, while potentially more accurate, operate as “black boxes,” making it difficult to discern the specific factors driving their predictions. This lack of transparency of machine learning models can hinder clinical decision-making and limit the potential for developing targeted interventions. Techniques like Shapley analysis have been proposed to help explain which of the features used by the machine learning model contributed most to a specific prediction, but these techniques don’t help explain how a model makes decisions in general.

In contrast, models based on single or very few mechanistic features derived from established physical laws and principles, are inherently more interpretable and explainable. This transparency helps engineers, scientists, and clinical decision makers understand the underlying phenomena and gain trust in the model’s predictions. Also, since mechanistic features are grounded in physical reality they capture fundamental relationships that hold across a range of systems and conditions. This inherent generalizability means they can often be extrapolated to scenarios outside the training data with more confidence than purely data-driven features. This is especially valuable when dealing with limited or expensive data collection.

The pathology of high-risk coronary plaques is well understood, with luminal narrowing and the presence of thincap-fibroatheroma (TCFA) as primary risk factors. Plaque rupture can be understood as a biomechanical process that occurs when the stress in the fibrous cap exceeds the tissue’s strength. Lumen geometry, plaque composition and morphology, and hemodynamics are all key factors that influence these stresses. Therefore, a biomechanical assessment of coronary lesions that incorporates this information into a cohesive, mechanistic model may provide further improvements in the ability to identify high-risk plaques that are likely to become the culprit lesions of ACS.

Here, we investigate the potential clinical utility of a novel, single mechanistic feature that integrates the underlying biomechanical characteristics of plaque rupture. This provides clinicians with a clear understanding of why a particular lesion is flagged as high-risk. The results in the present work are derived using data from the EMERALD study and compared to models previously evaluated.

## 2 Methods

Detailed statistical methods are presented in the Supplementary Appendix.

### 2.1 Study population

The present study was conducted using data from the EMERALD study (NCT02374775). The study retrospectively enrolled patients from 11 international cardiovascular centers across 5 countries that had undergone coronary CTA between 1-24 months prior to experiencing a clearly documented case of acute coronary syndrome (ACS). ACS was defined as either acute myocardial infarction (MI) or unstable angina with objective evidence of plaque rupture as verified by invasive coronary angiography. Exclusion criteria included ACS related to in-stent restenosis lesions, secondary MI due to other general medical conditions, a previous history of coronary artery bypass graft surgery, inadequate coronary CTA image quality. In addition, 3 of the 72 patients and 7 of the 216 lesions considered in the original EMERALD study were excluded due to a failure to perform the computational biomechanical analysis. In the present study, a total of 69 patients and 209 lesions were considered.

### 2.2 Analysis of coronary CTA and invasive coronary angiography results

Coronary CTA images were analyzed for Adverse Plaque Characteristics (APC) and quantitative plaque volumes at core laboratories in Seoul National University Bundang Hospital and HeartFlow, Inc., respectively. Lesions exhibiting more than 30% diameter stenosis (DS) based on coronary CTA assessment were selected for APC analysis. An independent observer, who was blinded to clinical data, evaluated the presence of APC (low-attenuation plaque, positive remodeling, napkin-ring sign, and spotty calcification) in each lesion according to established definitions. Angiography images were assessed in a blinded fashion at a core laboratory (Seoul National University Hospital) in order to identify the culprit lesion for ACS. Accordingly, each lesion was assigned a binary label as either culprit of ACS or nonculprit.

### 2.3 Analysis of hemodynamic parameters by CFD

CFD was used to evaluate the hemodynamic forces acting on the internal surface of each lesion using the methods described in Taylor et al. (10). As was done for the EMERALD study, the solution to the CFD problem was post-processed to compute four hemodynamic parameters to be used in prediction models: (1) ${\mathrm{FFR}}_{\mathrm{CT}}$; (2) change in ${\mathrm{FFR}}_{\mathrm{CT}}$ across the lesion ($\Delta {\mathrm{FFR}}_{\mathrm{CT}}$); (3) wall shear stress (WSS) (7, 11, 13); and (4) axial plaque stress (APS).

### 2.4 Analysis of biomechanical parameters by computational solid mechanics

**Figure 1:**
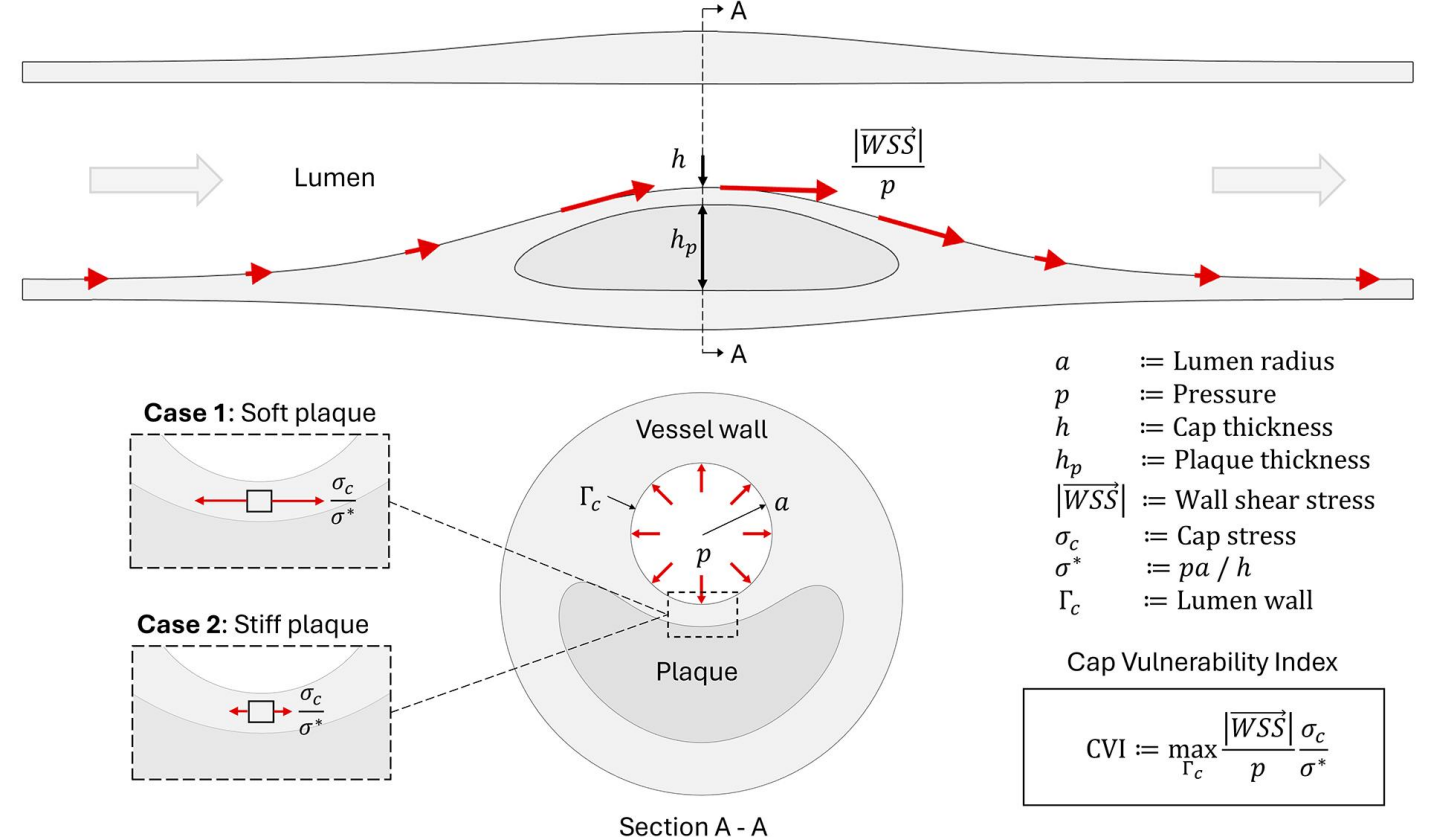
An illustration of variables included in calculation of the CVI parameter. Note that in the case of a soft plaque the cap stress is expected to be greater since the underlying plaque contributes less to the support of the circumferential stress. Similarly, in the case of an elevated wall shear stress which would be expected to lead to thinning of the cap, this would lead to an increase in CVI.

Computational solid mechanics was used to estimate stresses in the fibrous cap for each lesion. All analyses were performed in a blinded fashion using an elastic model derived from CT images. Briefly, lesion models were constructed by isolating a sub-volume of the CT image, including the vessel wall and surrounding heart tissues. *In-vivo* elastic properties were estimated according to the local Hounsfield Unit (HU), and hemodynamic tractions obtained from the CFD analysis were used as loading. The deformation of the vessel wall was computed by solving a linear elasticity problem with homogeneous Dirichlet boundary conditions. After the deformation field was obtained, the solution was post-processed to compute membrane stresses at the lumen boundary which were used to compute biomechanical metrics quantifying the stress state in the fibrous cap which was assumed to uniformly cover the entire lesion. The following cap properties were assumed in the computation: Elastic modulus, $E_{c}=200$ kPa, Poisson’s ratio $\nu_{c}=0.25$, and cap thickness h=20μm. A single biomechanical parameter, denoted by Cap Vulnerability Index (CVI), was considered. Figure 1 is a schematic depicting the relevant variables for calculation of CVI for a lesion.

CVI was computed as follows:

$$\text{CVI}=\max_{\Gamma_{c}}\frac{\sigma_{c}}{\sigma^{*}}\frac{\left\|WSS\right\|}{p},$$ (1)

where $\sigma^{*}$ is the local critical (thin-walled cylinder) stress limit, defined as:

$$\sigma^{*}=\frac{\;p\;a}{h},$$ (2)

where p is the local hemodynamic pressure, h is the assumed cap thickness, a is the local lumen radius, and $\Gamma_{c}$ is the surface of the fibrous cap.

**Figure 2:**
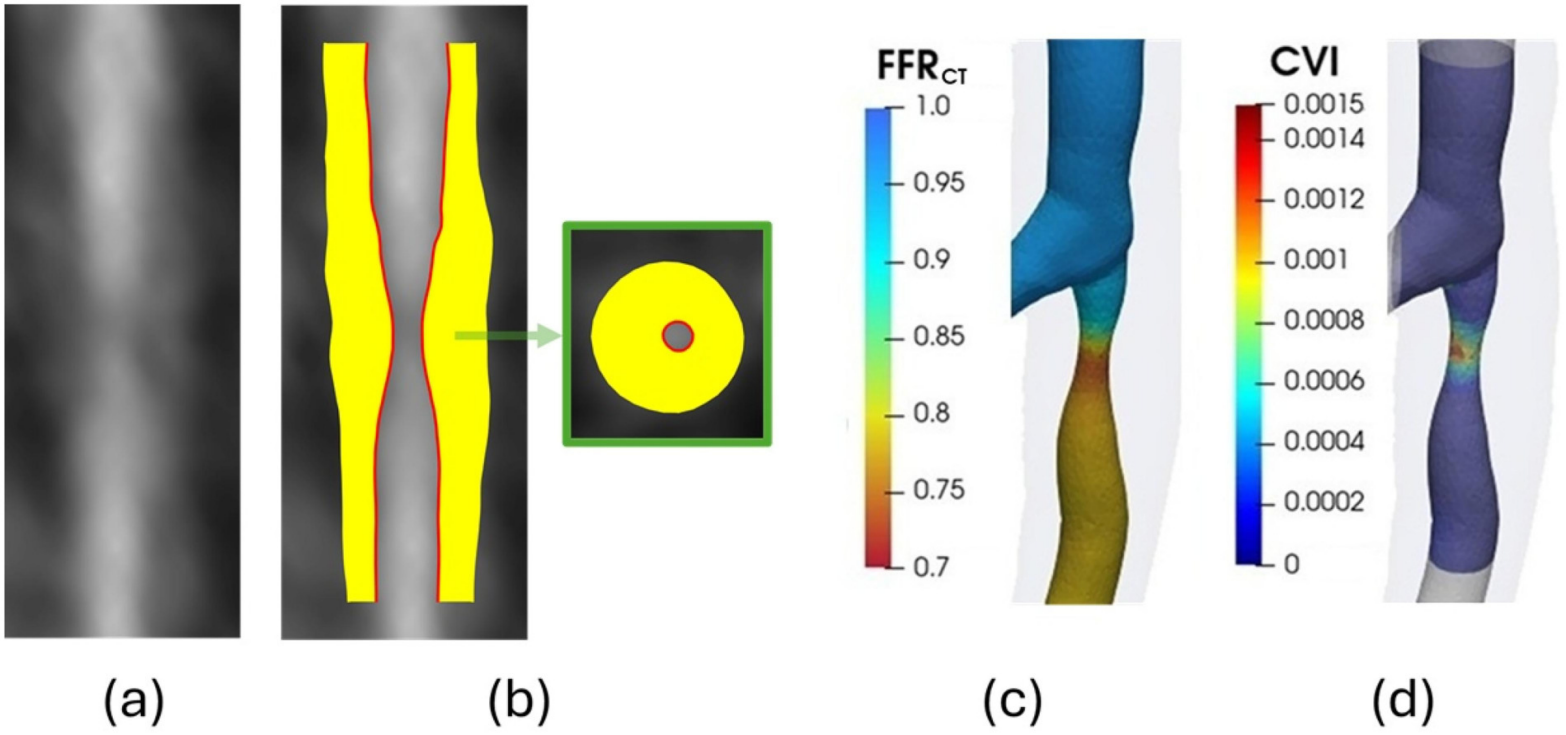
An example for the analysis of a lesion depicting **(a)** straightened CPR image of the lumen and plaque, **(b)** segmented inner and outer wall and noncalcified plaque in yellow, **(c)** ${\mathrm{FFR}}_{\mathrm{CT}}$ over the segmented region, **(d)** CVI parameter.

Figure 2 depicts the cCTA image data, ${\mathrm{FFR}}_{\mathrm{CT}}$ and CVI for a lesion.

### 2.5 Statistical analysis

All statistical analyses were conducted on a per-lesion basis using the features in Table 1.

**Table 1:** Definitions of the 11 lesion metrics considered in this study.

Parameter	Definition
Lesion geometry	
Diameter stenosis (DS)	Minimum lumen diameter relative to proximal reference lumen diameter
Lesion length (L)	Length of the vessel centerline between lesion endpoints
Qualitative plaque characteristics	
Low attenuation plaque (LAP)	Plaque with a pixel <=30 HU
Spotty calcification (SC)	Average density of > 130 HU and diameter <3 mm in any direction
Positive remodeling (PR)	Remodeling index of >=1.1
Napkin ring sign (NRS)	Ring-like attenuation form with peripheral high and central lower attenuation portion
Hemodynamic parameters	
Fractional flow reserve (FFR)	The ratio of mean downstream coronary pressure to the aortic pressure
ΔFFR	The difference of FFR between lesion endpoints
Wall shear stress (WSS)	Average hemodynamic shear stress acting on vessel wall
Axial plaque stress (APS)	Average hemodynamic axial stress acting on vessel wall
Biomechanical parameters	
Cap vulnerability index (CVI)	Maximum value of $\frac{\sigma_{c}}{\sigma^{*}}\frac{\left\|WSS\right\|}{p}$

First, each feature was analyzed independently for its ability to discriminate between culprit and nonculprit lesions using logistic regression and the AUC performance metric. The AUC for CVI was compared to the AUCs of the other independent features previously established in the EMERALD I study. The AUC metric was computed using Leave-pair-out cross-validation (LPOCV) instead of the 5-fold cross validation used in the EMERALD I study. For details on LPOCV, see Supplementary Appendix. Additionally, all features were ranked using a Shapley analysis, as described in Supplementary Appendix. Each feature’s ΔAUC value was determined by computing its Shapley value, and the ΔAUC for CVI was compared to the values for the other EMERALD I features.

Next, the CVI prediction model was compared to a multi-feature model trained with the 10 features from the EMERALD I study. The comparison was conducted using a bootstrap method to establish a confidence interval for the AUCs of the two models. Bootstrapping was performed by sampling the dataset with replacement in order to compute 1,000 replicates of the AUC for each model.

Finally, an optimal cutoff value for CVI was determined using ROC analysis, as described in Supplementary Appendix. The cutoff value was used to partition the set of lesions into two groups: those with CVI greater than the cutoff, and those below the cutoff. The two groups were then analyzed using Kaplan-Meier survival analysis to test for statistical differences in cumulative risk for ACS.

## 3 Results

### 3.1 Baseline characteristics of patients and lesions

**Figure 3:**
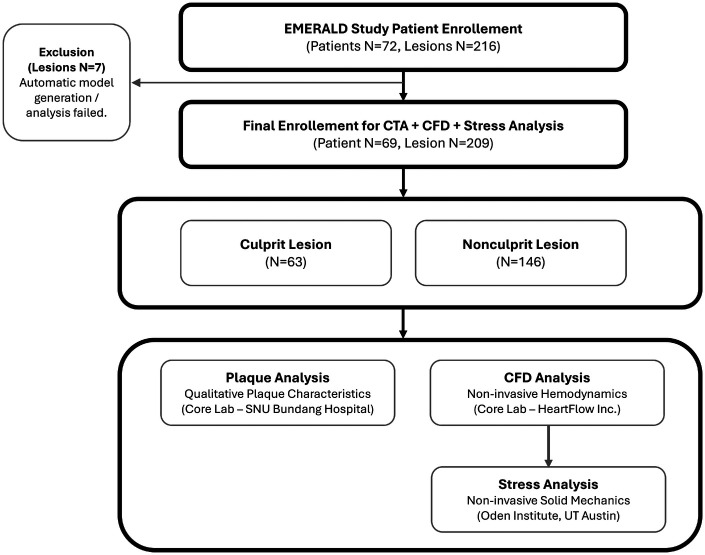
Study flow.

A total of 69 ACS patients with 63 culprit and 146 nonculprit lesions were included in the present analysis (Figure 3). Additional details of the EMERALD I clinical study pertaining to inclusion/exclusion criteria, data collection, analysis, and results on qualititative plaque and quantitative hemodynamic features can be found in the original publication (6).

### 3.2 Comparison of anatomical severity, plaque, hemodynamic, and structural analysis parameters between culprit and nonculprit lesions

Table 2 presents the parameters investigated in the current analysis for both the culprit and nonculprit lesions. Among the geometric features (qualitative percent diameter stenosis and lesion length) percent diameter stenosis was significantly higher for culprit lesions compared to nonculprit lesions (p<0.001). Culprit lesions showed a higher frequency of low-attenuation plaque, positive remodeling, napkin-ring sign, and spotty calcification than nonculprit lesions, with LAP, PR, and NRS being significantly higher in the culprit lesions (p<0.001). In terms of hemodynamic parameters $\Delta {\mathrm{FFR}}_{\mathrm{CT}}$ and WSS were significantly higher in the culprit lesions whereas ${\mathrm{FFR}}_{\mathrm{CT}}$ and APS showed no significant difference between culprit and nonculprit lesions. Lastly, CVI was significantly larger in culprit lesions (mean CVI 0.511±0.790 in culprit lesions vs. 0.131±0.220 in nonculprit lesions).

**Table 2:** Reported values are mean ± std. and frequency (percentage) for continuous and categorical features, respectively.

Parameter	Nonculprit lesion	Culprit lesion	
	(*n*=146)	(*n*=63)	*P* value${}^{a}$
Lesion geometry			
DS	0.432 ± 0.151	0.552 ± 0.147	<0.001
L	15.31 ± 8.06	17.86 ± 7.73	0.034
Adverse plaque characteristics			
LAP	11 (7%)	19 (30%)	<0.001
SC	29 (19%)	26 (41%)	0.002
PR	16 (10%)	22 (34%)	<0.001
NRS	13 (8%)	21 (33%)	<0.001
Hemodynamic parameters			
FFR	0.865 ± 0.135	0.846 ± 0.110	0.285
ΔFFR	0.066 ± 0.091	0.170 ± 0.163	<0.001
WSS	139.0 ± 78.1	206.3 ± 101.1	<0.001
APS	924 ± 2776	2126 ± 3776	0.026
Biomechanical parameters			
CVI	0.131 ± 0.220	0.511 ± 0.790	<0.001

${}^{a}$Welch’s t-test and chi-squared test were used to compare culprit vs. nonculprit lesions for continuous and categorical features, respectively.

### 3.3 Single feature models for the prediction of ACS

Table 3 presents the AUC values for each single feature model. The top performing single feature for discrimination between culprit vs. nonculprit lesions using the proposed logistic regression model is the biomechanical parameter, CVI, with an AUC score of 0.752. This result suggests the potential of this metric as an independent predictor for ACS, and demonstrates the potential clinical utility of quantification of cap stress by CCTA stress analysis. The two worst performing metrics are lesion length and spotty calcification identified qualitatively from CCTA. In the original EMERALD I analysis, lesion length was also shown to have lower information gain compared to other features that are more predictive of ACS such as $\Delta {\mathrm{FFR}}_{\mathrm{CT}}$, %DS, WSS, Remodeling Index, and low Hounsfield Units. Conversely, features associated with soft plaque such as NRS, PR, and LAP demonstrated moderate values (0.622, 0.620, and 0.613). The best performing hemodynamic feature is $\Delta {\mathrm{FFR}}_{\mathrm{CT}}$, which agrees with the original findings of the EMERALD I study (6) (AUC=0.712).

**Table 3:** AUC scores for each feature obtained using logistic regression and LPOCV.

Feature	AUC
CVI	0.752
ΔFFR	0.712
WSS	0.692
DS	0.690
NRS	0.622
LAP	0.669
PR	0.620
APS	0.617
FFR	0.609
SC	0.607
L	0.593

### 3.4 CVI for predicting future ACS events

**Figure 4:**
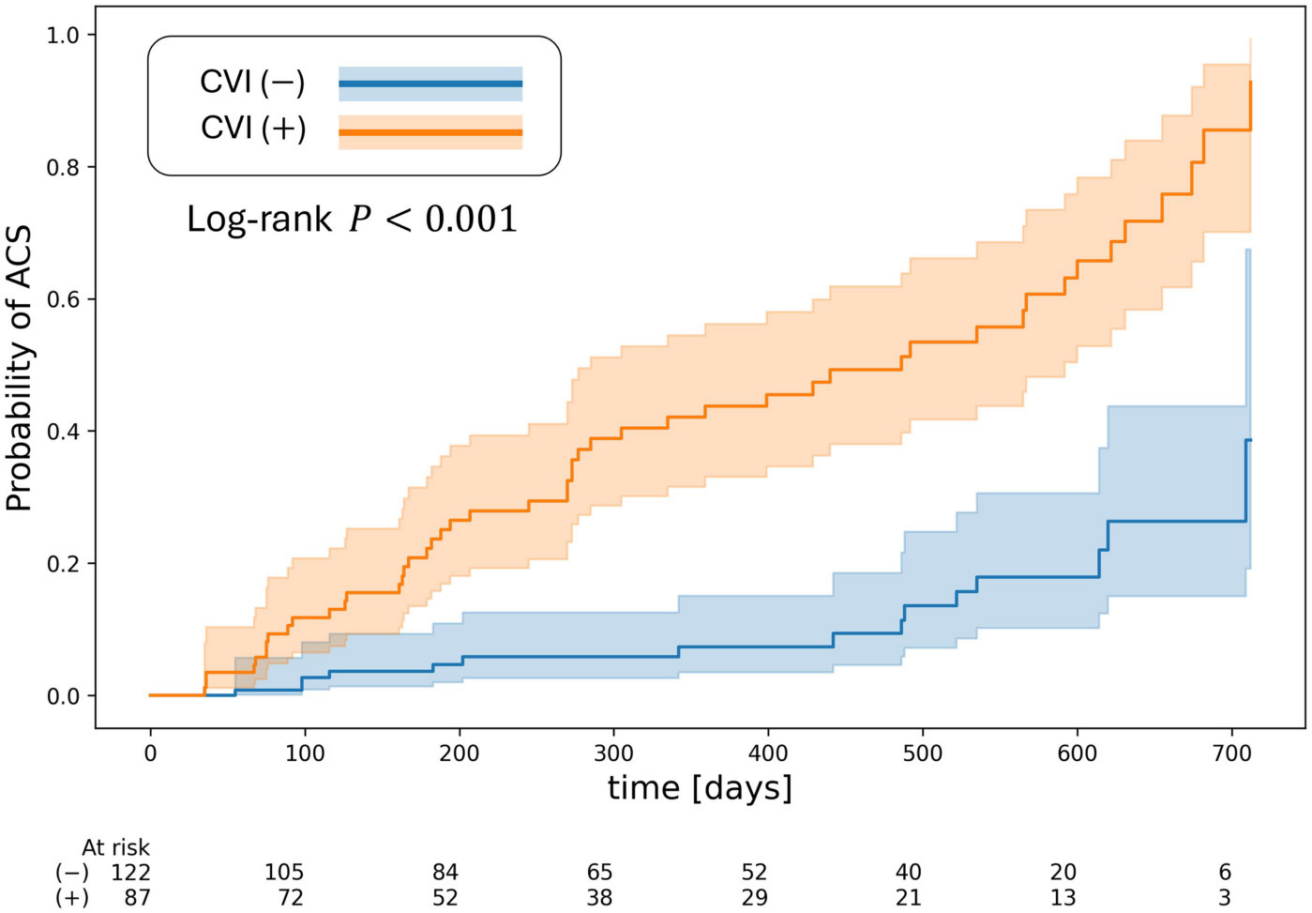
Kaplan-Meier.

Figure 4 shows the Kaplan-Meier risk stratification of having ACS based on the quantitative CVI metric over the duration of the EMERALD I study, approximately 2 years. CVI (−) and CVI(+) were defined based on the theshold of CVI identified using Youden’s J statistic. Lesions in the CVI(+) group were shown to have a statistically higher probability of having ACS within 2 years compared to CVI(-) lesions as determined by the log-rank test (p<0.0001).

### 3.5 Δ AUC for evaluating feature importance

**Figure 5:**
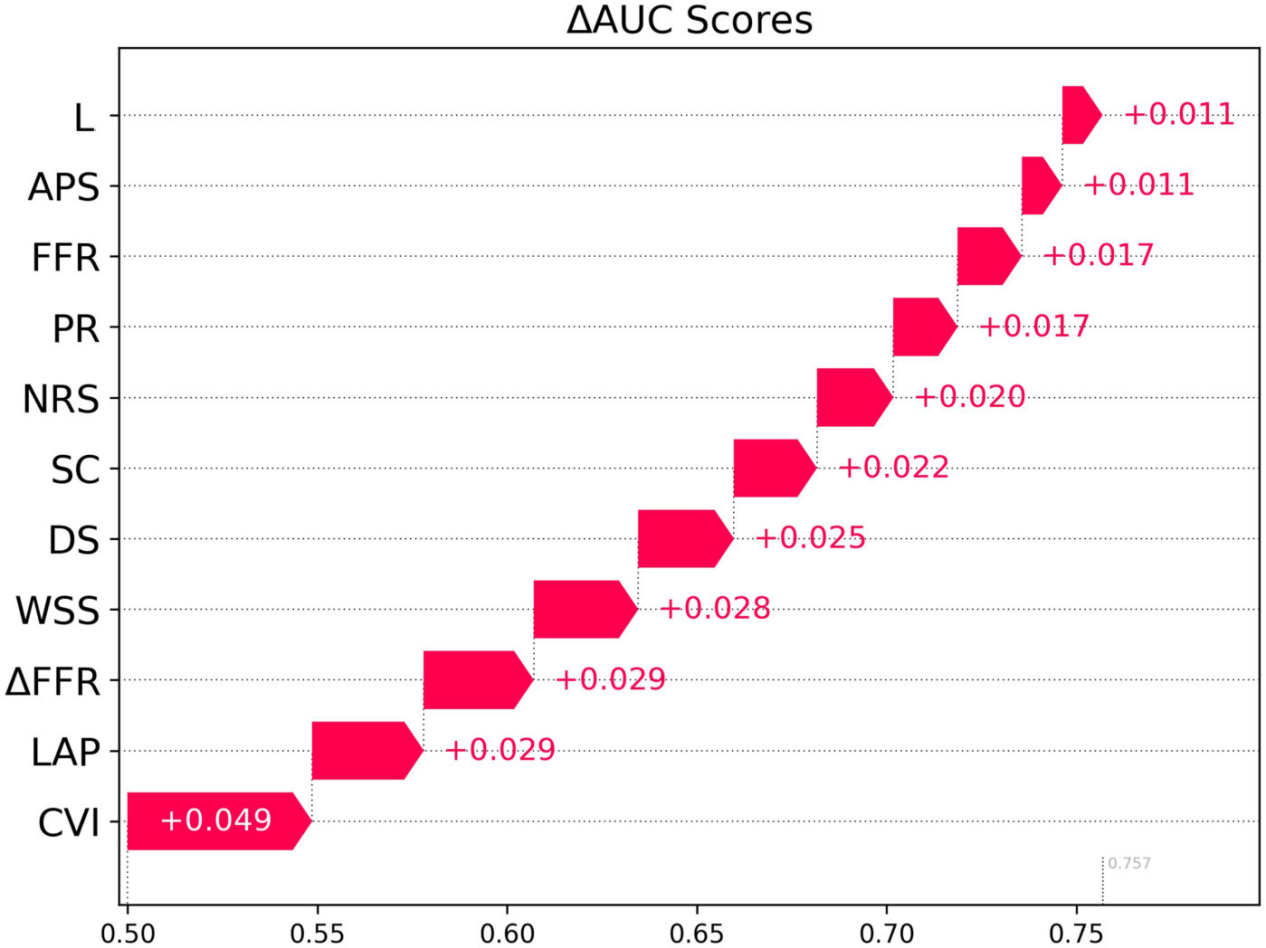
Shapley analysis demonstrating relative importance of all 11 features when included in a combined model.

To evaluate importance of the individual metrics, all features were included in a combined 11-feature model. ΔAUC scores were then computed for each feature and ranked according to the magnitude of ΔAUC. The ΔAUC scores are shown in Figure 5. The features with the highest contribution to the total AUC are CVI (ΔAUC 0.049), LAP (ΔAUC 0.029) and $\Delta {\mathrm{FFR}}_{\mathrm{CT}}$ (ΔAUC 0.029).

### 3.6 Comparison between CVI metric and models from EMERALD I

**Figure 6:**
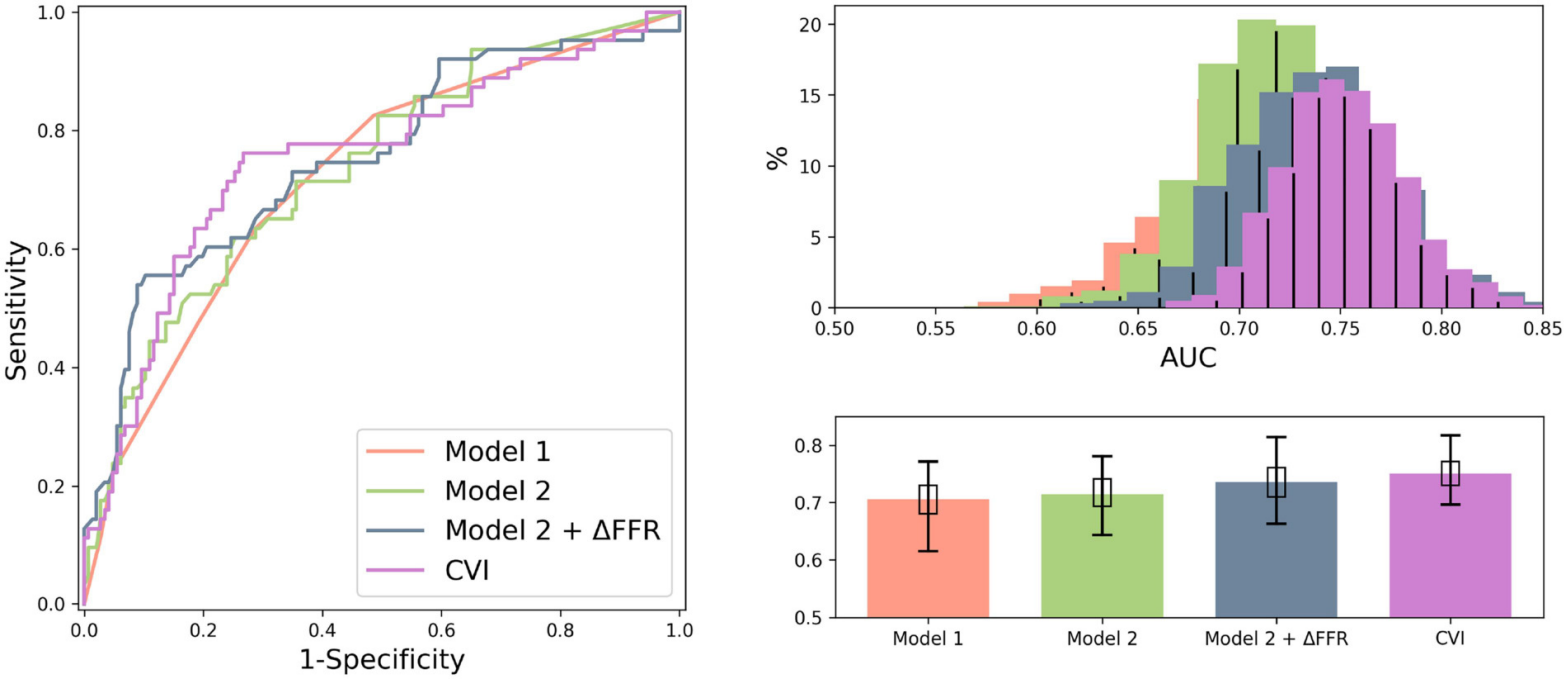
Comparison between CVI and EMERALD I models using a bootstrapping method to estimate the AUC for each model. Note that CVI, as an individual parameter, performed as well as the best model from EMERALD I, i.e., Model 2+$\Delta {\mathrm{FFR}}_{\mathrm{CT}}$.

To evaluate the utility of the CVI metric to predict risk of ACS, we then compared this measure to the multi-feature models from the original EMERALD I study. As shown in Table 4 and Figure 6, CVI, as an individual metric performed as well as the best model from EMERALD I, i.e., Model 2+$\Delta {\mathrm{FFR}}_{\mathrm{CT}}$ but this latter model includes 7 distinct variables.

**Table 4:** CVI vs. EMERALD I Models. AUC values for each model were computed using LPOCV. Model 1 was based upon the anatomic variables diameter stenosis and lesion length. Model 2 added the Adverse Plaque Characteristics to Model 1, Model 3 added the hemodynamic parameters listed in Table 2 to Model 2.

Model	AUC
CVI	0.752
EMERALD I (Model 2+ΔFFR)	0.752
EMERALD I (Model 3)	0.750
EMERALD I (Model 2)	0.737
EMERALD I (Model 1)	0.722

## 4 Discussion

The present study investigated the utility of noninvasive biomechanical assessment using CTA to extract lumen geometry and coronary plaques, computational fluid mechanics to quantify pressure and shear forces acting on the lesions and computational solid mechanics to compute stress in the plaques themselves in order to identify coronary plaques that caused ACS. A new metric, the Cap Vulnerability Index (CVI) was defined in an effort to extract a meaningful parameter from the biomechanical model related to the balance between cap stress and cap strength and hence could be hypothesized to be related to plaque rupture.

The CVI index yielded the the highest contribution to the AUC and emerged as the strongest independent predictor in discriminating between culprit vs. nonculprit lesions. As demonstrated herein, this single measure matched the performance of the best statistical model from EMERALD I which included 7 variables. These results support the importance of integrating noninvasive biomechanical assessment of coronary lesions for improving the ability to identify vulnerable plaques prior to rupture.

In the present study, we analyzed coronary lesions using coronary CTA scans performed before clearly documented ACS events (most were acute MI) to identify the strongest predictors of ACS. Features associated with the presence of soft plaque and high cap stress were the stongest predictors of ACS.

### 4.1 Study limitations

The current study has several limitations. First, as in the case of the primary study using this dataset (6), all data was collected and analyzed retrospectively. Thus, the prognostic performance of this approach cannot be ascertained for prospective analysis of CCTA for a given patient. Since all patients enrolled had ACS, the data analysis was necessarily performed at the plaque level. Thus patient-level factors that could predispose a patient to having ACS could not be evaluated in this study. Second, there was no control group of patients that did not have an event. Thus, the predictive performance of the methods described herein is unknown for lower risk cohorts of patients. Third, there were no separate derivation or validation cohorts due to the small sample size. Fourth, the only patient data included in the analysis was the CCTA image data. Fifth, since it is not possible to ascertain the fibrous cap thickness or elastic modulus, these values were assumed based on literature values. Finally, the results of this study cannot be extrapolated to the general population of patients with asymptomatic atherosclerotic coronary disease without an external control population.

## 5 Conclusions

Patient-specific biomechanical models of coronary lesions can be constructed using cCTA data to extract lumen geometry and atherosclerotic plaques, computational fluid mechanics to estimate forces acting on plaques and computational solid mechanics to compute stresses in the plaques. Stresses computed from such integrated models may improve identification of potential culprit lesions for ACS and simplify the interpretation of risk factors for individual lesions.

## Data Availability

The raw data supporting the conclusions of this article will be made available by the authors, without undue reservation.
