# Supplementary material for: Biomechanical index for predicting the risk of acute coronary syndrome
Source: Front Cardiovasc Med. 2026 Mar 24;13:1766059. doi: 10.3389/fcvm.2026.1766059 (PMC13053513; doi:10.3389/fcvm.2026.1766059)
Supplement: Supplementary file 1 [file Supplementaryfile1.pdf]

## Appendix A.

### *Appendix A.1. Leave-pair-out cross-validation (LPOCV)*

The AUC for a prediction model is interpreted as the probability that the model correctly ranks a culprit lesion as having a higher probability of ACS than a nonculprit lesion. To estimate the AUC, we use leave-pair-out cross-validation (LPOCV) which gives an *almost* unbiased estimate as described by Airola et al. in [11].

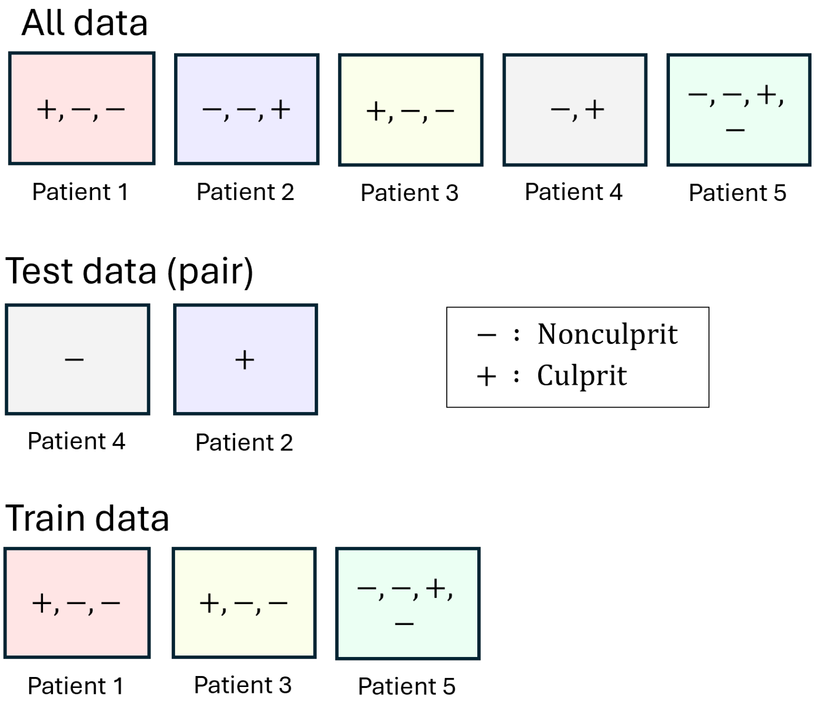


Supplementary Figure 1: A simple example demonstrating Leave-pair-out Cross Validation (LPOCV).

With LPOCV, the data is split into testing and training sets by a single culprit-nonculprit pair. The model is trained on all data excluding lesions with the same patient IDs as the pair, and then tested on the pair. This process is repeated for all culprit-nonculprit pairs in order to get a single estimate of the AUC. The formula for computing the AUC with LPOCV is

given by:

$$\text{AUC} = \frac{1}{|\boldsymbol{X}^+||\boldsymbol{X}^-|} \sum_{\boldsymbol{x}_i \in \boldsymbol{X}^+} \sum_{\boldsymbol{x}_j \in \boldsymbol{X}^-} H\left(p_{\boldsymbol{Z}_{ij}}(\boldsymbol{x}_i) - p_{\boldsymbol{Z}_{ij}}\left(\boldsymbol{x}_j\right)\right), \tag{A.1}$$

where $H$ is the Heaviside step function; $\boldsymbol{Z}_{ij} = \{(\boldsymbol{x}_k, y_k, g_k) \ : \ g_k \neq g_i \wedge g_k \neq g_j\}$ is the subset of $\boldsymbol{D}$ excluding all samples with patient IDs $G_i$ or $G_j$; and $\boldsymbol{X}^+$ and $\boldsymbol{X}^-$ are the subsets of $\boldsymbol{X}$ corresponding to culprit ($y_i = 1$) and nonculprit ($y_i = 0$) lesions, respectively. A simple example of LPOCV is illustrated in Figure 1. Note, we explicitly prevent lesions belonging to the same patient from being included in both testing and training sets.

To construct an ROC curve using LPOCV, we use the tournament LPOCV algorithm described in [12].

*Appendix A.2. Shapley Analysis*

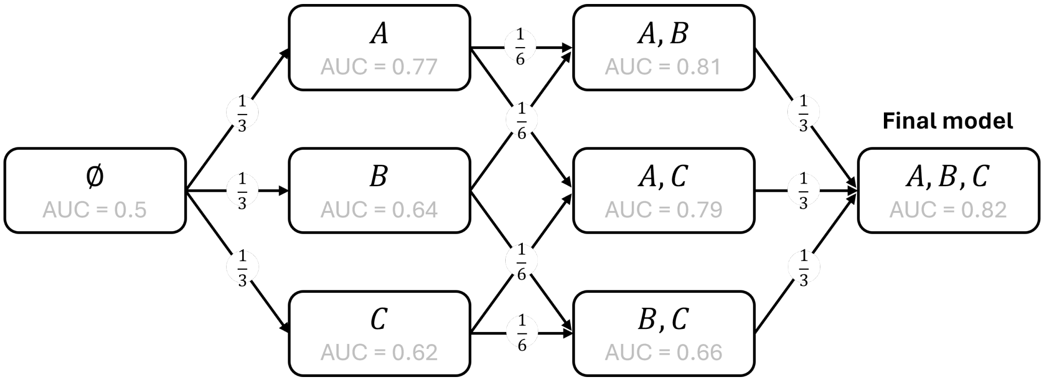


Supplementary Figure 2: A simple example illustrating how to estimate the additive predictive value of a class of features.

Feature ranking was performed by considering each feature's expected marginal increase to the AUC, denoted by ΔAUC. This was done by computing the Shapley value. To demonstrate how this is calculated, consider the simple example illustrated in Figure 2. Let $A$, $B$, and $C$ denote three features. Suppose we want to quantify the contribution of feature $A$. First, we form all possible subsets of the other features, $B$ and $C$, including the empty set. Then, we compute the change in AUC for each subset as a result of adding $A$. Finally, we take a weighted average over all subsets to determine the expected increase in AUC for feature $A$. The weights are

determined such that the sum of the ΔAUC scores for each feature equal the total AUC value corresponding to the model containing all features. For the example in Figure 2, this is calculated as follows:

$$\Delta\text{AuC}\,(A) = \frac{1}{3}(0.77-0.5)+\frac{1}{6}(0.81-0.64)+\frac{1}{6}(0.79-0.62)+\frac{1}{3}\,(0.82-0.66) = 0.20. \tag{A.2}$$

This calculation is then repeated for the other features. In general, for a set of features, $F$, the ΔAUC scores for a feature $f \in F$ is given by the following formula:

$$\Delta\text{AUC}\,(f) = \sum_{S\in\mathcal{P}(F\setminus\{f\})} \left(|S|\binom{|F|}{|S|}\right)^{-1} (\text{AUC}\,(S\cup\{f\}) - \text{AUC}\,(S)), \tag{A.3}$$

where $\mathcal{P}(\cdot)$ returns the powerset, and AUC$(\cdot)$ returns the AUC for a model with a given set of features.

*Appendix A.3. Optimal Cutoff*

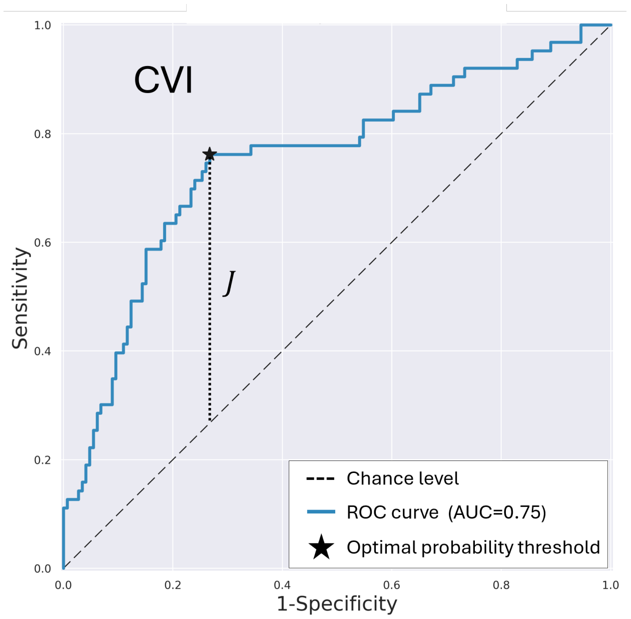


Supplementary Figure 3: Youden's $J$ statistic on the ROC curve.

The optimal cutoff threshold was determined using logistic regression and ROC analysis. To demonstrate this procedure, let us consider CVI

as an example. The logistic model was fit once using all samples, and the false positive rate (FPR) and true positive rate (TPR) were computed as a function of a probability threshold, $t$. Then, an optimal probability threshold was computed by maximizing Youden's J statistic:

$$J(t) := TPR(t) - FPR(t). \tag{A.4}$$

Graphically, the optimal threshold corresponds to the probability that maximizes the distance between the chance line and the ROC curve, as shown in Figure 3. After the optimal probability threshold is determined, the logistic function in equation (**??**) is inverted to solve for the optimal cutoff value for CVI.

*Appendix A.4. Feature Correlations*

Since CVI includes the magnitude of the wall shear stress, CVI is expected to be correlated with WSS and ΔFFR-CT. However, the wall shear stress and pressure represent merely the force acting on the plaque, whereas CVI is based on a stress analysis that includes unique plaque morphology and properties (derived from the Hounsfeld units of the CT exam). Figure 4 shows a correlation analysis for the features considered in this study.

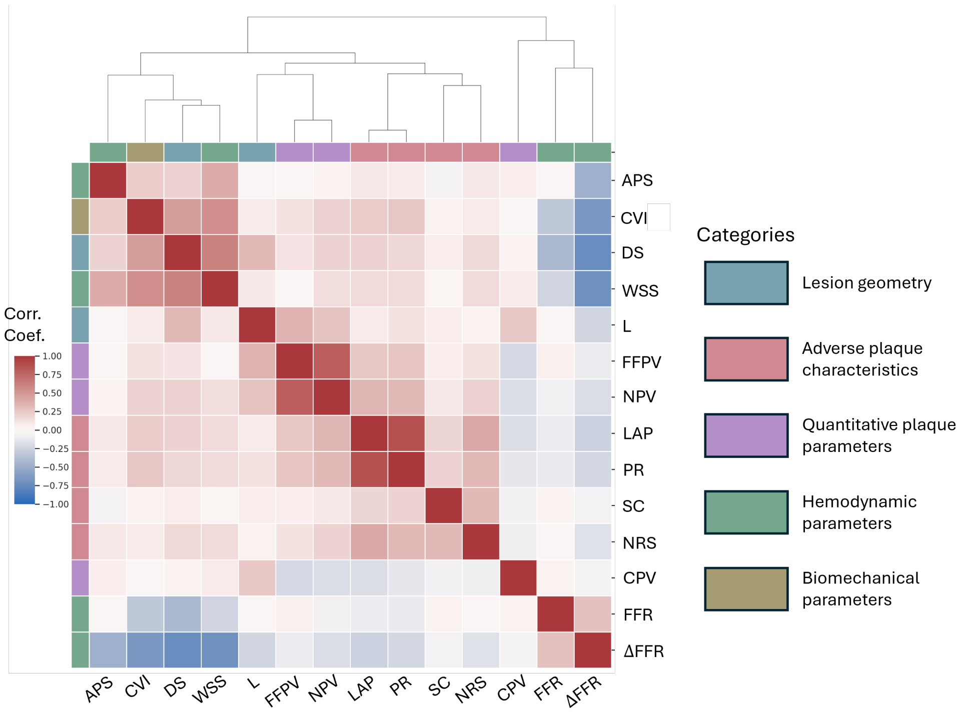


Supplementary Figure 4: Correlation coefficients
